# Prevalence and Risk Factors of Childhood Obesity at a Hospital in Cuenca, Ecuador

**DOI:** 10.7759/cureus.97163

**Published:** 2025-11-18

**Authors:** Cindy Calle, Jorge Diaz

**Affiliations:** 1 Faculty of Medicine, Universidad del Azuay, Cuenca, ECU

**Keywords:** bmi, childhood obesity, enobi, metabolic syndrome, paediatric endocrinology, paediatrics, prevalence, risk factors

## Abstract

Introduction: Childhood obesity has become increasingly prevalent and represents an important public health concern. This study aimed to determine the prevalence of childhood obesity, identify associated risk factors, and describe related clinical findings in a pediatric population in Cuenca, Ecuador.

Methods: A cross-sectional, observational, and analytical study was conducted with 644 patients who attended the outpatient clinic of Hospital Humanitario Fundación Pablo Jaramillo Crespo between July 2020 and June 2021. Data were obtained from the HPJC electronic medical records and processed using an original questionnaire. Prevalence rates, obesity classifications, and associations with risk factors were analyzed using odds ratios (OR). The presence of metabolic syndrome and related clinical signs was also evaluated.

Results: Of the total sample, 43.17% (n=278) were boys and 56.83% (n=366) were girls. The highest prevalence of childhood obesity occurred in the 12-18-year age group (41.25%). The overall OBI prevalence was 12.4%, with class I obesity being the most frequent (70%). The strongest associated risk factors were family history of obesity (OR 7.91; 95%CI 4.5-13.90), daily consumption of pastries and/or sugary drinks (OR 8.38; 95%CI 4.94-14.20), and snacking between meals and/or skipping meals (OR 8.71; 95%CI 4.78-15.91). Metabolic syndrome was present in 36.96% of patients. The most frequent clinical findings were increased abdominal circumference (73.75%), abdominal striae (43.75%), hypertriglyceridemia (43.75%), and hypercortisolemia (36.36%).

Conclusions: Comprehensive assessment of pediatric patients with attention to modifiable risk factors, particularly dietary habits, physical activity, and screen time, is essential. Early intervention targeting these factors may reduce the prevalence and complications of childhood obesity.

## Introduction

Childhood malnutrition has long been recognized as a major global health challenge. Traditionally, undernutrition predominated in developing countries, whereas obesity was more frequent in developed nations. However, globalization and changing food systems have blurred this distinction, leading to a worldwide rise in obesity across all socioeconomic settings [1]. Moreover, lifestyle disruptions during the COVID-19 pandemic further exacerbated unhealthy dietary habits and reduced physical activity, reinforcing the need for a comprehensive, multidimensional approach to prevention.

Obesity, defined as a body mass index (BMI) at or above the 95th percentile for age and sex based on CDC growth charts, has shown a steady global increase from 4.8% in 1990 to 6.2% in 2015 [2]. In the United States, prevalence rates are estimated at 13.7% among preschool children, 18.7% among school-aged children, and 20.6% among adolescents. Cross-country comparisons remain challenging due to methodological and definitional differences; nevertheless, surveys indicate prevalence rates exceeding 30% in most countries across North and South America, as well as in England, Italy, Portugal, and Spain, while rates in some European countries and China remain closer to 15% [3].

In Latin America and the Caribbean, data from the Pan American Health Organization (PAHO) and the Food and Agriculture Organization (FAO) reveal a similar upward trend. Among children under five years of age, the prevalence of overweight and obesity increased from 6.6% to 7.2%, with several countries, including Argentina, Brazil, Chile, Costa Rica, Mexico, and Ecuador, reporting rates above the regional average [2].

In Ecuador, the 2015 ENSANUT (Encuesta Nacional de Salud y Nutrición) survey reported overweight and obesity prevalence rates of 8.6% among children under five years and 26% among adolescents aged 12-18 years. The subsequent 2018 ENSANUT survey, focusing on children aged 5-11 years, showed a further increase to 35.4%, up from 31.3% in 2015. The provinces with the highest reported rates include Chimborazo, Cotopaxi, Bolívar, El Oro, and Azuay [4].

Obesity constitutes a major public health challenge in Ecuador. In 2014, approximately USD 4.3 million of the national health budget was allocated to nutrition-related initiatives [5]. Beyond its economic impact, the rising prevalence of obesity is accompanied by an alarming increase in comorbidities. Conditions once considered exclusive to adults, such as hypertension, type 2 diabetes mellitus, and non-alcoholic steatohepatitis, are now being diagnosed at progressively younger ages. Consequently, obesity demands coordinated short- and long-term public health interventions [6].

Although numerous international studies have explored the determinants of childhood obesity, evidence from Andean or Ecuadorian pediatric populations remains scarce, particularly concerning the effects of pandemic-related behavioral changes on obesity risk. This study addresses this gap by providing region-specific data and identifying contextually relevant, modifiable risk factors.

## Materials and methods

This was an analytical cross-sectional study conducted at Hospital Humanitario Fundación Pablo Jaramillo Crespo (HPJC), Cuenca, Ecuador. The study was approved by the Ethics Committee of the University of Azuay, Cuenca, Ecuador, and authorization to access patient records was granted by the director, HPJC. A waiver of informed consent was approved, as no identifiable personal data was collected.

Study population

Pediatric patients aged 2-18 years who attended the pediatric outpatient clinic between July 1, 2020, and June 30, 2021, comprising 2,695 medical records, were included in the study. Exclusion criteria included medical conditions that interfered with accurate anthropometric or dietary assessment (e.g., severe edema, physical disability, or chronic growth disorders), syndromic or endogenous obesity, and pregnancy in adolescents. These conditions can lead to weight gain through distinct pathophysiological mechanisms, producing falsely elevated BMI values that could compromise the validity of the findings. Patients without a legal guardian to provide consent were also excluded. 

**Figure 1:**
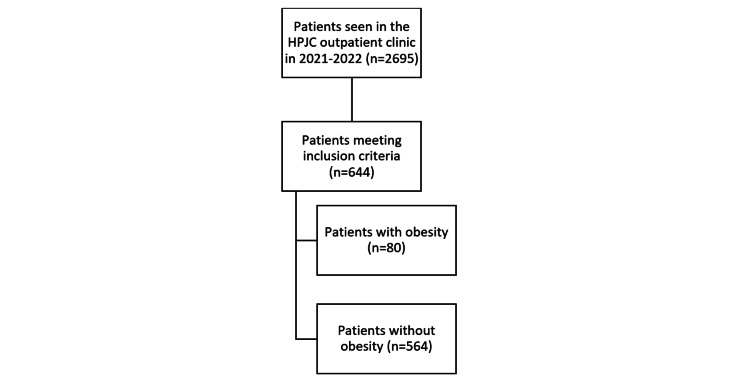
Selection of the study participants HPJC: Hospital Humanitario Fundación Pablo Jaramillo Crespo

Consecutive non-probabilistic sampling was applied to include all patients who met predefined eligibility criteria during the study period. Eligibility was confirmed through electronic medical record review and verified by the attending pediatrician to ensure consistent application of inclusion and exclusion criteria. After screening, 644 patients were included in the final analysis, of whom 80 met criteria for childhood obesity. Applying identical selection criteria to all consecutive eligible patients minimized selection bias and improved representativeness of the outpatient pediatric population. The selection process is summarized in Figure 1.

Objectives

The primary objective was to determine the prevalence of childhood obesity and assess its association with potential risk factors in the pediatric population. Secondary objectives included identifying the prevalence of metabolic syndrome and related comorbidities.

Data collection and tool

Following authorization, anonymized data were extracted from the HPJC Medical System database for all pediatric outpatient visits during the study period. Data were subsequently filtered by International Classification of Diseases, Tenth Revision (ICD-10) diagnosis codes: E66 (“Obesity”), E66.0 (“Obesity due to excess calories”), E66.9 (“Obesity, unspecified”), and R63.5 (“Abnormal weight gain”). Patients with overlapping diagnostic codes were excluded to prevent duplication. Information was organized using a self-developed structured data collection form named ENOBI (ENcuesta de OBesidad Infantil), developed in Google Forms (Google LLC, Mountain View, California, United States), which captured demographic, clinical, and analytical variables (see Appendices).

The data collection form consisted of three sections: (i) medical history, focusing on prenatal, natal, and postnatal risk factors, (ii) classification of obesity among diagnosed patients; and (iii) analytical parameters for evaluating complications. To ensure diagnostic correlation, data on related ICD-10 codes (E30.8, E30.9, E30, and E78.0) were also retrieved.

Anthropometric measurements (weight, height, and BMI) were obtained by two trained HPJC nurses following WHO standardized procedures. Instruments included a digital scale and stadiometer, both calibrated daily. The scale was verified with certified 1 kg and 5 kg weights (accuracy ±0.1 kg), and the stadiometer was checked using a 1 m calibration rod (accuracy ±0.1 cm). Inter-observer reliability was excellent (intraclass correlation coefficient >0.9 for both measurements). Laboratory and clinical assessments were conducted blinded to obesity status to minimize measurement bias.

Data analysis

Data collected via Google Forms were exported to Excel Plus 2019 (Microsoft Corporation, Redmond, Washington, United States), where descriptive statistics and prevalence calculations were performed using pre-defined formulas. Variables included overall obesity prevalence, obesity grades, metabolic syndrome (for patients >10 years), and clinical and analytical findings. To ensure data quality, the form was pilot-tested with 25 records to assess clarity and consistency, and all entries were cross-checked with HPJC electronic medical records. Both investigators received standardized training on anthropometric measurement and data entry procedures.

Associations between obesity and potential risk factors were analyzed using odds ratios (OR) and 95% confidence intervals (CIs). Continuous or multi-category variables were dichotomized based on established clinical cutoffs from the literature. Statistical analyses were performed in IBM SPSS Statistics for Windows, version 27 (IBM Corp., Armonk, New York, United States), using a significance level of p < 0.05. Multivariable logistic regression models adjusted for potential confounders, including age and sex, to avoid bias.

## Results

The total study sample comprised 644 children, with 564 children without obesity and 80 children with obesity. The total sample and the group with children without obesity showed a predominance of children aged 3-6 years, representing 24.22% and 26.6%, respectively. In contrast, among children with obesity, the most represented age group was 12-18 years (41.25%). Children aged 2-3 years were the least represented among patients with obesity (3.75%), despite being the third most frequent age group in the general outpatient population. Regarding sex distribution, female children predominated across all subgroups, with an average of approximately 56%, as summarized in Table 1.

**Table 1:** Distribution of children of the different categories according to age and sex

Characteristics	Total Children (n=644)		Children With Obesity (n=80)		Children Without Obesity (n=564)	
	Frequency	Percentage	Frequency	Percentage	Frequency	Percentage
Age Group						
2 to 3 years	132	20.50	3	3.75	129	22.87
3 to 6 years	156	24.22	6	7.50	150	26.60
6 to 10 years	145	22.52	25	31.25	120	21.28
10 to 12 years	106	16.46	13	16.25	93	16.49
12 to 18 years	105	16.30	33	41.25	72	12.77
Sex						
Male	278	43.17	35	43.75	243	43.09
Female	366	56.83	45	56.25	321	56.91

Primary results

**Figure 2:**
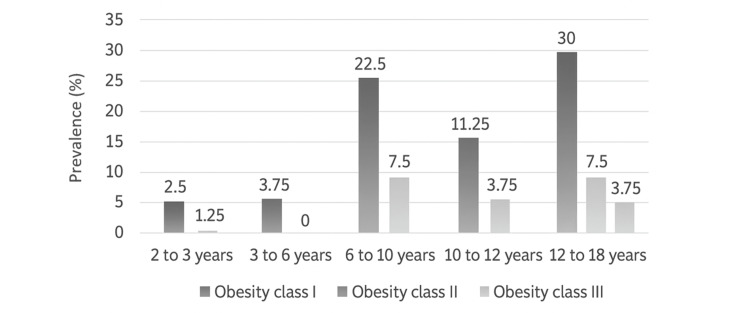
Prevalence of obesity according to age group Data given as percentages

Obesity diagnosis was based on BMI-for-age percentiles and classified according to the following categories: class I (BMI ≥ 95th percentile), class II (BMI ≥ 120% of the 95th percentile or ≥ 35 kg/m², whichever was lower), and class III (BMI ≥ 140% of the 95th percentile or ≥ 40 kg/m², whichever was lower). The overall prevalence of obesity was 12.4% (95% CI: 9.9-15.0%), with class I being the most frequent (70%, 95%CI: 60.0-80.0%), followed by class II (20%, 95%CI: 11.2-28.8%) and class III (10%, 95%CI: 3.4-16.6%). When stratified by sex, female children predominated in class I (45%; 95% CI: 33.9-55.9%), whereas male children predominated in class II (12.5%; 95%CI: 5.2-19.8%) and class III (6.25%; 95%CI: 0.9-11.5%). Obesity prevalence by age group is shown in Figure 2.

Metabolic syndrome criteria were consistently applied to all patients over 10 years of age, using age-appropriate cutoffs according to established pediatric guidelines. Among the 46 children with obesity aged more than 10 years, 17 met the diagnostic criteria for metabolic syndrome (36.96%; 95%CI: 23.1-50.9%), with a predominance of female sex (58.82%; 95%CI: 35.4-82.2%).

Several risk factors were significantly associated with obesity. The strongest associations were found for a family history of chronic non-communicable diseases (OR 7.91; 95%CI: 4.5-13.9), daily consumption of pastries and/or sugary drinks (OR 8.38; 95%CI: 4.94-14.2), and snacking between meals and/or skipping main meals (OR 8.71; 95% CI: 4.78-15.91). Other factors, such as cesarean birth (OR 0.96; 95%CI: 0.60-1.54) and rural residence (OR 0.71; 95%CI: 0.40-1.27), showed weaker or non-significant associations. These results suggest that certain lifestyle behaviors-particularly excessive sugar intake and irregular eating patterns-may increase the risk of obesity by nearly eightfold. The details are presented in Table 2.

**Table 2:** Risk factors associated with obesity in children

Variables	Children With Obesity (n=80), n	Children Without Obesity (n=564), n	Odds Ratio	95% Confidence Intervals
Family history of pathological conditions (high BMI, DM and/or HTN)	63	180	7.91	4.50 – 13.90
Abnormal weight gain during pregnancy	14	78	1.32	0.71 – 2.47
< 3 prenatal ultrasounds	7	33	1.54	0.66 – 3.62
< 5 prenatal checkups	11	27	3.17	1.51 – 6.68
Cesarean delivery	38	273	0.96	0.61 – 1.54
Preterm or post-term birth	14	72	1.45	0.77 – 2.71
Low or excessive weight gain at birth	25	135	1.44	0.87 – 2.41
Small or large for gestational age	19	129	1.05	0.61 – 1.82
Inadequate or insufficient intake of fruits, vegetables, and/or macronutrients	28	153	1.45	0.88 – 2.37
Daily consumption of pastries and/or sugary drinks	58	135	8.38	4.94 – 14.20
Snacking between main meals and/or skipping meals	66	198	8.71	4.78 – 15.91
Insufficient physical activity	50	234	2.35	1.45 – 3.81
≥ 2 hours per day of screen time	68	354	2.36	1.38 – 4.03
Insufficient daily sleep hours	31	114	2.50	1.52 – 4.09
< 7 average academic performance in the last school year	15	66	1.74	0.94 – 3.23
Basic/primary education level of parent or guardian	26	147	1.37	0.82 – 2.26
Living in small and/or rented housing	34	129	2.49	1.53 – 4.05
Rural residence area	16	147	0.71	0.40 – 1.27

Secondary results

Physical examination findings among patients with obesity included abdominal circumference ≥ 90th percentile (73.75%; 95%CI: 64.45-83.05), acanthosis nigricans (32.5%; 95%CI: 22.24-42.76), lower abdominal striae (43.75%; 95%CI: 32.71-54.79), pubertal development alterations (40%; 95%CI: 29.06-50.94), blood pressure ≥ 90th percentile (2.5%; 95%CI: −0.28-5.28), and goiter (1.25%; 95%CI: −1.18-3.68).

Complementary laboratory assessments revealed high prevalences of elevated triglycerides (43.75%; 95%CI: 32.71-54.79%), low high-density lipoproteins (HDL) (42.5%; 95%CI: 31.49-53.51%), and elevated daytime plasma cortisol in patients older than 15 years (36.36%; 95%CI: 25.60-47.12%). Less frequent findings included anemia (1.25%; 95%CI: −1.18-3.68%) and bone age alterations on carpogram (5%; 95%CI: 0.16-9.84%). Additional laboratory and clinical results are detailed in Table 3.

**Table 3:** Prevalence of abnormal paraclinical results in patients with obesity (N=80)

Paraclinical Test	Frequency (Percentage)	95% CI
Decreased hemoglobin (< 11 g/dl)	1 (1.25)	-1.18 - 3.68
Decreased hematocrit (< 32%)	1 (1.25)	-1.18 - 3.68
Altered fasting blood glucose		
– In < 12 years (≥ 110 mg/dl)	7 (15.22)	5.07 - 19.43
– In ≥ 12 years (≥ 100 mg/dl)	11 (32.35)	22.10 - 42.60
Elevated glycated hemoglobin (≥ 5.7%)	11 (13.75)	6.20 - 21.30
Elevated basal insulin for age (≥ p95)	25 (31.25)	21.37 - 41.13
Altered diurnal plasma cortisol		
– In < 15 years (< 24.0 > 230 ng/ml)	7 (10.14)	3.84 - 16.43
– In ≥ 15 years (< 24.0 > 290 ng/ml)	4 (36.36)	25.60 - 47.12
Elevated TSH for age (≥ p95)	5 (6.25)	1.36 - 11.14
Decreased FT4 for age (≤ p5)	3 (3.75)	-0.41 - 7.91
Elevated TGO or TGP for age (≥ p95)	21 (26.25)	16.75 - 35.75
Elevated total cholesterol for age (≥ p95)	17 (21.25)	12.20 - 30.30
Elevated LDL cholesterol for age (≥ p95)	14 (17.50)	9.10 - 25.90
Decreased HDL cholesterol (< 40 mg/dl)	34 (42.50)	31.49 - 53.51
Elevated triglycerides (≥ 110 mg/dl)	35 (43.75)	32.71 - 54.79
Abnormal liver ultrasound findings	26 (32.50)	22.24 - 42.76
Abnormal bone age X-ray	4 (5)	0.16 - 9.84

All variables were complete for all participants; thus, no missing data handling or imputation methods were required in the analyses.

## Discussion

Pathophysiology, risk factors, and clinical assessment of childhood obesity

Obesity is a chronic, non-communicable disease characterized by excessive accumulation of body fat resulting from a sustained positive energy balance, which adversely affects health [7,8]. In children over two years of age, diagnosis is based on BMI adjusted for age and sex. According to the CDC, obesity corresponds to a BMI ≥95th percentile in individuals aged 2-20 years [3]. The WHO defines it as BMI >3 SD for children under five and >2 SD for those aged 5-19 years [9, 10, 11]. The summary can be found in Table 4.

**Table 4:** Classification of childhood obesity

Based on Cause	
Type	Pathogenesis
Nutritional/exogenous (95–99%)	Multifactorial (genetic, environmental, psychosocial)
Organic/endogenous (1–5%)	Endocrinopathies (hypothyroidism, hypopituitarism, Cushing’s syndrome); CNS lesions, infections, and tumors; insulinomas; genetic syndromes
Based on BMI	
Category	Definition (CDC)
Underweight	BMI < 5th percentile
Normal weight	BMI ≥ 5th percentile and < 85th percentile
Overweight	BMI ≥ 85th percentile and < 95th percentile
Obesity (Class I)	BMI ≥ 95th percentile
Severe obesity (Class II)	BMI ≥ 120% of 95th percentile or ≥ 35 kg/m² (whichever is lower)
Severe obesity (Class III)	BMI ≥ 140% of 95th percentile or ≥ 40 kg/m² (whichever is lower)

Obesity arises from complex interactions among neural, hormonal, and metabolic signals that regulate appetite and energy expenditure through hypothalamic pathways [12,13]. Energy balance depends on basal metabolic rate, thermogenesis, and physical activity. Excess caloric intake promotes adipocyte hyperplasia and hypertrophy, leading to insulin resistance, hypoxia, and low-grade inflammation. Adipose tissue then overproduces leptin, cytokines, and angiotensinogen while reducing adiponectin, contributing to ectopic fat deposition and metabolic dysfunction.

In children, adipocyte hyperplasia predominates initially, preserving normal physiology. However, progression to hypertrophy leads to adipocyte dysfunction and central obesity, explaining why some obese children remain metabolically healthy while others develop early cardiometabolic risk [12,14].

Early diagnosis is critical to prevent complications and guide interventions. Assessment begins with a detailed history, including personal and family medical history (e.g., type 2 diabetes, dyslipidemia, cardiovascular disease), and evaluates prenatal, perinatal, and postnatal risk factors [15,16]. Maternal smoking during pregnancy increases obesity risk by up to 50% due to fetal vasoconstriction and hypoxia [9,17]. Both preconceptional and gestational obesity, as well as gestational diabetes, alter the intrauterine environment, causing fetal anatomical and physiological changes [18]. Maternal preeclampsia is associated with higher BMI in offspring during puberty, while early maternal menarche predisposes to accelerated growth in the first two years of life.

Perinatal risk factors include prematurity, small or large size for gestational age, and macrosomia, all of which predispose to insulin resistance. Rapid postnatal weight gain in these infants further increases risk. In contrast, breastfeeding exerts a protective effect by regulating satiety through leptin-mediated mechanisms. Early weaning has been associated with a fourfold increase in obesity risk, whereas each additional month of breastfeeding reduces risk by approximately 4% up to nine months [15,17,19].

Dietary habits play a key role in pediatric obesity. Between 10% and 27% of children consume sugary drinks, fast food, or snacks more than three times per week [17]. A single soft drink can supply up to 15% of daily caloric needs, and excessive salt intake increases food consumption through osmotic mechanisms. Irregular meal patterns-such as skipping breakfast, large portions, or reduced family meals-further promote early adiposity rebound [3,9].

Sedentary behavior, defined as insufficient physical activity and excessive screen exposure (>1 hour/day for ages 2-4 and >2 hours/day for those >5 years), contributes significantly to obesity. Physical inactivity reduces basal metabolic rate, diet quality, and sleep duration, disrupting leptin and ghrelin regulation [3,17,20].

Adolescence represents a critical period for obesity persistence; approximately 80% of obese adolescents remain obese into adulthood. The hypothalamic peptide kisspeptin, which increases leptin secretion once lean mass reaches a critical threshold, influences appetite, metabolism, and glucose homeostasis. Early menarche, associated with higher adiposity, may further reinforce this cycle [21,22].

Clinical evaluation should include blood pressure, BMI, waist circumference, and, when available, tri-ponderal mass index (TMI, kg/m³), which may better estimate body fat in children aged 8-18 years [23]. Skinfold thickness at triceps and subscapular sites also provides useful indicators [24]. Laboratory evaluation typically includes fasting glucose, insulin, HbA1c, lipid profile, and liver enzymes, with additional endocrine tests as clinically indicated [9,25].

Pediatric metabolic syndrome occurs primarily in the context of obesity and requires at least three of the following: central obesity, dyslipidemia, hypertriglyceridemia, hypertension, or glucose intolerance. In Ecuador, the NHANES III cutoffs are commonly applied for children aged 10-12, whereas the ALAD criteria are used for adolescents [26,27]; see Table 5 for complications [28,29].

**Table 5:** Complications of obesity

System	Complications
Cardiovascular	Hypertension (HTN), dyslipidemia, structural heart disease, heart failure, premature atherosclerosis
Endocrine	Prediabetes/Type 2 diabetes, hyperandrogenism, polycystic ovary syndrome, metabolic syndrome, early/late puberty onset
Gastrointestinal	Non-alcoholic fatty liver disease, cholelithiasis
Nutritional	Vitamin D and iron deficiency
Musculoskeletal	Genu varus, genu valgum, fractures, musculoskeletal pain, femoral epiphysiolysis
Dermatological	Furunculosis, hidradenitis suppurativa, intertrigo, acanthosis nigricans
Psychological	Low self-esteem, body dissatisfaction, depression, anxiety, eating behavior disorders
Other less frequent	Asthma, obstructive sleep apnea, chronic kidney disease

**Figure 3:**
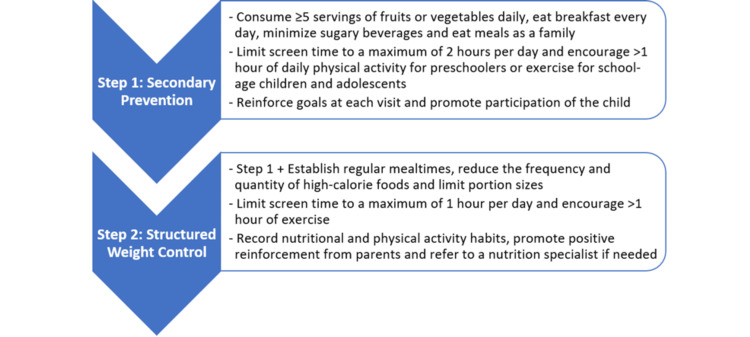
Steps in the management of obesity Image Credit: Authors

Management follows a stepwise approach, emphasizing education and prevention at the primary care level. Objectives include optimizing weight, sleep, and physical activity, defined as any skeletal muscle movement that increases energy expenditure, or structured exercise designed to improve fitness. Interventions should be individualized and adjusted to disease severity, with specialist referrals for comorbidities as needed, as illustrated in Figure 3 [20,30,31].

The COVID-19 pandemic has significantly impacted lifestyle. Quarantine increased screen time, consumption of baked goods, decreased sleep, and reduced physical activity [32,33]. Obesity also exacerbates COVID-19 risk by promoting a pro-inflammatory, prothrombotic, and immunosuppressive state [34].

Comparison of study findings with the literature

López-Sobaler et al., in the ALADINO (ALimentación, Actividad Física, Desarrollo INfantil y Obesidad) 2019 study of the Spanish pediatric population, reported that 40.6% of children presented excessive weight gain, with 17.3% classified as obese [35]. Similarly, Monciño Sandoval, in a retrospective cross-sectional study in Mexico City, found a prevalence of approximately 29% [36], reinforcing that obesity affects both developed and developing nations. 

In Ecuador, Montoya and Martínez (2021) reported a prevalence of 10.5% [37], closely aligning with the 12.4% found in this study. It is inferred that younger children (<10 years) attended outpatient clinics more frequently, likely due to parental concern; however, adolescents, while comprising a smaller proportion of total visits, exhibited the highest rates of obesity. This finding is consistent with Coronado Pérez, who analyzed pediatric obesity in Panama (2015-2019) and reported a peak prevalence between 11-12 years (33%) [38]. This trend may reflect greater exposure to modifiable lifestyle risk factors among adolescents in recent years.

Although the present study found a slightly higher obesity prevalence in female children, several international and national studies report a higher prevalence in male children (57.1% vs. 48.9%) [37,38]. These differences may reflect behavioral or sociocultural factors rather than biological sex, as no consistent evidence supports sex as a biological determinant of obesity.

Regarding classification, Class I obesity was the most prevalent across all ages, suggesting that most cases are detected at early stages. The predominance of more advanced obesity classes (II and III) among adolescents highlights the progressive nature of this condition over time. Additionally, the COVID-19 quarantine period likely exacerbated sedentary behavior and unhealthy eating habits, influencing these trends.

Both modifiable and non-modifiable risk factors were associated with varying degrees of influence on obesity development. Prenatal and perinatal factors showed minimal impact; although most presented ORs >1, the findings were inconclusive. Yáñez-Ortega et al. reported obesity rates of 15.8% in preterm and 44.4% in post-term patients in Spain, with 10% of children with obesity delivered via cesarean section [39]. A notable exception was family history; Montoya V. and Martínez J. found that 30.55% of obese patients had a family history of obesity, diabetes, or hypertension [37], emphasizing the importance of inherited and environmental familial habits.

Among postnatal modifiable factors, the strongest correlations were found for daily consumption of pastries or sugary drinks, snacking between meals, sedentary behavior, and excessive screen time. Cobo Bonilla identified poor diet (OR 7), sedentary lifestyle (OR 2.1), and skipping breakfast (OR 5.4) as significant contributors to exogenous obesity in their study in Quito [40]. Similarly, Urbano Arcos and Beltrán Gómez found that more than two hours of screen exposure increased obesity risk dramatically (OR 230) [41]. These results align with the present findings and may explain the surge in pediatric obesity observed during quarantine.

Socioeconomic factors also influenced obesity risk. In the current study, rural residence appeared protective (OR 0.71), possibly due to healthier eating patterns and greater physical activity. In contrast, Serral et al. reported that low socioeconomic status increased obesity risk (OR 1.6) [42]. Such discrepancies may reflect regional differences in lifestyle, access to processed foods, and cultural habits.

Physical examination findings supported the predominance of central (android) obesity, evidenced by increased abdominal circumference. Cruz Sosa reported similar results, with 83.3% of overweight and obese patients exceeding the 90th percentile for waist circumference [43]. Other characteristic findings, such as acanthosis nigricans and abdominal striae, reflected early physiopathological changes of obesity; in the current study, 32.5% and 43.75% of patients, respectively, presented these features. Pubertal alterations were also frequent (40%) in our study, suggesting potential links with precocious puberty.

Although hypertension and goiter were infrequent in this cohort, Coronado Pérez found that 34.48% of children with obesity had systolic blood pressure above the 95th percentile [38], indicating that such complications may develop later. The most common laboratory alterations in this study, hypertriglyceridemia, low HDL, steatosis, and hypercortisolemia, are key components of insulin resistance and metabolic syndrome. Approximately one in three obese patients in the present study met the diagnostic criteria for metabolic syndrome, mirroring the 36% prevalence reported by Coronado Pérez [38]. Similarly, Agüero et al. demonstrated that 100% of patients with metabolic syndrome had underlying overweight or obesity [44], underscoring the pivotal role of obesity in metabolic comorbidity development.

The low frequency of other complications, such as altered bone age and hypothyroidism, supports the notion that obesity-related comorbidities develop progressively over time.

Limitations and recommendations

This study used secondary data extracted from electronic medical records. Although the data were complete and the inclusion/exclusion criteria were strictly applied, reliance on retrospective records introduces a potential for information bias due to inconsistent documentation. Additionally, because the study population was drawn from a single outpatient clinic, generalizability to broader pediatric populations may be limited.

Future studies are recommended to employ direct, prospectively collected data, ideally using the ENOBI tool developed for this study. Such studies should maintain a cross-sectional design but aim to assess both the prevalence and severity of overweight and obesity across different age groups, as this information remains scarce in Ecuador. Clinically, it is recommended that all obesity consultations include comprehensive documentation of risk factors, physical examination findings, and disease progression. Routine use of the ENOBI form could standardize data collection and enhance understanding of obesity severity. Following these recommendations may facilitate early identification of modifiable risk factors, improve prevention strategies, and reduce the burden of obesity-related complications in the pediatric population.

## Conclusions

Childhood obesity has emerged as a highly prevalent and multifactorial health condition, characterized not only by excessive weight gain but also by a constellation of metabolic disturbances that may progress to metabolic syndrome. In this study, a prevalence of 12.4% was identified for childhood obesity, most strongly associated with a family history of obesity, frequent consumption of sugary foods and beverages, and irregular eating habits such as skipping main meals. These findings reflect the growing burden of pediatric obesity, an issue further exacerbated by pandemic-related lifestyle changes.

By comprehensively assessing both affected and non-affected children and emphasizing modifiable postnatal factors such as diet, physical activity, and screen exposure, this study highlights the critical importance of early identification and intervention. Clinically, implementing targeted, evidence-based prevention strategies could substantially reduce obesity prevalence, limit disease progression, and prevent long-term metabolic complications. Overall, the results underscore the urgent need for proactive, multidisciplinary approaches in pediatric care and public health policy. Future research should focus on longitudinal and community-based studies to evaluate the sustained effects of targeted interventions and to elucidate additional socio-environmental determinants contributing to childhood obesity.

## Appendices

Study questionnaie (ENOBI)

**Table 6:** Childhood Obesity Survey (ENOBI)

SECTION 1: ANAMNESIS	
Category	Options
Patient information	
How old is the patient?	Older infant >2 y <3 / Preschooler 3 a <6 / School-age 6 a <10 / Preadolescent 10 a <12 / Adolescent 12 a 18
What is the patient´s sex?	Male / Female
Family history	
Do first- and/or second-degree relatives have overweight or obesity?	Yes / No
Do first- and/or second-degree relatives have diabetes mellitus (DM)?	Yes / No
Do first- and/or second-degree relatives have hypertension (HTN)?	Yes / No
Do first- and/or second-degree relatives have heart disease?	Yes / No
What is the representative’s height?	_____
What is the representative’s weight?	______
Prenatal personal history	
How was the weight gain during pregnancy?	Low / Normal / Excessive
How many obstetric ultrasounds were performed during pregnancy?	< 3 / ≥ 3
How many prenatal check-ups were performed?	< 5 / ≥ 5
Natal personal history	
What was the type of delivery?	Cesarean / Vaginal
What was the gestational age (GA) of the patient?	Preterm (<37 weeks) / Term (37 - 41.6 weeks) / Post-term (≥ 42 weeks)
What was the birth weight?	Normal (2500 – 3500 g) / Abnormal (< 2500 o > 3500 g)
Neonatal anthropometry	Small / Normal / Large
Postnatal personal history	
How many fruits does the patient eat?	No / Per week ___ / Per day ___
How many salads and/or vegetables?	No / Per week ___ / Per day ___
Does the patient eat protein daily?	No / Per week ___ / Per day ___
Does the patient consume dairy products daily?	No / Per week ___ / Per day ___
Does the patient consume sugary drinks more frequently than unsweetened ones?	No / Per week ___ / Per day ___
Does the patient consume industrial pastries more than once per week?	No / Per week ___ / Per day ___
Does the patient skip any of the main meals (breakfast, lunch, dinner)?	Yes (which one and why?) ______ / No
Does the patient snack between meals (excluding mid-morning and afternoon snacks)?	Yes / No
Does the patient engage in exercise or any type of physical activity?	Every day / ≤ 2 times per week / >2 times per week / Total time: _____
How many hours per day does the patient spend in front of screens (excluding academic hours)?	≥ 2 hours / < 2 hours
How many hours does the patient sleep per day?	4 - 8 hours / 8 - 12 hours / >12 hours
What is the average school performance (out of 10)?	< 7 (unsatisfactory) / 7 - 9 (good) / > 9 (outstanding)
Does the patient have a history of comorbidities related to metabolic syndrome (HTN, DM, dyslipidemia)?	Yes / No
Socioeconomic background	
What is the education level of the representative?	Primary / Secondary / Higher education
What is the area of residence?	Rural / Urban
What type of housing do they have?	House / Apartment / Room
SECTION 2: CLINICAL MARKERS	
Weight-for-age	Normal (between -2DS to +2DS) / Altered (between <2DS or >2DS)
Height-for-age	Normal (between -2DS to +2DS) / Altered (<2DS or >2DS)
Waist circumference-for-age	Normal (
BMI	Obesity Class I ≥p95 / Obesity Class II ≥120% of p95 or ≥35 kg/m2 (whichever is lower) / Obesity Class III ≥140% of p95 or ≥40 kg/m2 (whichever is lower)
Tri-ponderal index	Normal (between -2DS to +2DS) / Altered (<2DS or >2DS)
Presence of acanthosis nigricans	Yes / No
Presence of stretch marks	Yes / No
Blood pressure	Normal
Presence of goiter	Yes / No
Tanner stage	Stage 1 / Stage 2 / Stage 3 / Stage 4 / Stage 5
SECTION 3: ANALYTICAL MARKERS	
Hemoglobin	Normal (≥11 g/dl) / Altered (<11g/dl)
Hematocrit	Normal (≥32%) / Altered (<32%)
Fasting glucosa	< 12 years: Normal (<109 mg/dl) / Altered (≥110 mg/dl); >12 years: Normal (<99 mg/dl) / Altered (≥100 mg/dl)
Fasting insulin (by age)	Normal (< p95) / Altered (≥ p95)
Glycosylated hemoglobin (HbA1c)	Normal (< 5.7%) / Altered (≥ 5.7%)
Plasma cortisol (a.m.)	< 15 years: Normal (24 - 230 ng/ml) / Altered (< 24 or > 230 ng/ml); >15 years: Normal (24 - 290 ng/ml) / Altered (< 24 or > 290 ng/ml)
AST and ALT (by age)	Normal (< p95) / Altered (≥ p95)
TSH (by age)	Normal (< p95) / Altered (≥ p95)
Free T4 (by age)	Normal (≥ p5) / Altered (< p5)
Total cholesterol (by age)	Normal (< p95) / Altered (≥ p95)
HDLc	Altered (<40 mg/dl) / Normal (≥40 mg/dl)
LDLc (by age)	Normal (< p95) / Altered (≥ p95)
Triglycerides	Normal (<109 mg/dl) / Altered (≥110 mg/dl)
Ultrasound (by age)	Normal / Altered
Carpogram (radiograph of the carpal bones) by age	Normal / Altered
